# Remediation of Micro- and Nanoplastics by Membrane Technologies

**DOI:** 10.3390/membranes15030082

**Published:** 2025-03-05

**Authors:** Michał Bodzek, Piotr Bodzek

**Affiliations:** 1 Institute of Environmental Engineering, Polish Academy of Sciences, 41-819 Zabrze, Poland; 2 Faculty of Medical Sciences in Zabrze, Medical University of Silesia, 40-055 Katowice, Poland; piotr.bodzek@sum.edu.pl

**Keywords:** micro- and nanoplastics removal, membrane processes, ultra- and microfiltration, reverse osmosis membrane bioreactor, membrane fouling

## Abstract

Micro- and nanoplastics (NPs) cannot be completely removed from water/wastewater in conventional wastewater treatment plants (WWTPs) and drinking water treatment plants (DWTPs). According to the literature analysis, membrane processes, one of the advanced treatment technologies, are the most effective and promising technologies for the removal of microplastics (MPs) from water and wastewater. In this article, firstly, the properties of MPs commonly found in water and wastewater treatment and their removal efficiencies are briefly reviewed. In addition, research on the use of microfiltration (MF), ultrafiltration (UF), nanofiltration (NF), reverse osmosis (RO), and membrane bioreactors (MBR) for the remediation of MPs and NPs from water/wastewater is reviewed, and the advantages/disadvantages of each removal method are discussed. Membrane filtration is also compared with other methods used to remove MPs. Furthermore, the problem of membrane fouling by MPs during filtration and the potential for MPs to be released from the polymeric membrane structure are discussed. Finally, based on the literature survey, the current status and gaps in research on MPs removal by membrane technologies are identified, and recommendations for further research are made.

## 1. Introduction

Global plastics production will reach over 400 million tons in 2022 [1,2,3,4]. The creation, development, and use of plastics have harmful consequences for the environment. Only 32.5% of plastics are recycled, 42.6% are used for energy production, and the remaining plastics (24.9%) end up in landfills and the environment. Despite the long-standing introduction of recycling, reports indicate that the single use of plastics is only 50%, which contributes to the daily increase in environmental pollution [5].

Plastics degrade over long periods of time, breaking down into fine particles by physical fragmentation, bioremediation, or oxidative, hydrolytic, and thermal degradation, with photodegradation being the most common process in the environment [6,7,8].

Mostly plastic litter, MPs and NPs are found in virtually all aquatic media, from oceans to lakes and rivers [9]. MPs are defined as plastics less than 5 mm in size, and are classified as primary and secondary depending on their source [10]. Primary MPs include small particles that have been produced for commercial use, while secondary MPs are formed after fragmentation of larger plastic objects [11]. Although primary MPs ideally retain their size, typically between 0.1 and 5000 μm, they undergo further fragmentation to form NPs, whose particle size ranges from 1 to 100 nm. MPs and NPs released from primary and secondary sources are present in wastewater [12], surface and groundwater, tap and bottled water [13,14,15,16,17,18,19] (Table 1). A study in the Seine River in Paris reported MPs of 3–108 m^−3^ [20], while in a highly urbanized river in Chicago, USA 1.94–17.93 m^−3^ [21]. A study by Baldwin et al. [22] found plastics in 107 samples from 29 Great Lakes in the USA, with MPs ranging from 0.05 to 32 m^−3^. Other studies have found MPs not only in the aquatic environment, but also in air [23], food [24], soil [25] and sediment [26].

The most common plastics found in the environment are polypropylene (PP), polyethylene (PE), polystyrene (PS), polyvinyl chloride (PVC), polycarbonate (PC), polyamides (PA), polyester (PEs), and polyethylene terephthalate (PET). These are reversible thermoplastic polymers that are highly recyclable and can be repeatedly heated, cooled, and shaped [7,33]. PE, PP, PA, PES, PS, and PET are the six most commonly detected polymers in wastewater, with contents of 64.07%, 32.92%, 10.34%, 75.36%, 24.17%, and 28.90%, respectively [34]. The main sources of MPs are plastic articles, such as bags used for food packaging, bottles, and cutlery, in addition to the tire and textile industries, including synthetic clothing and textiles [35,36,37].

MPs vary in size, color, and shape. They most commonly take the shape of fibers, fragments, spheres (balls, pellets, and granules), foam, and film [38]. The four types of MPs most commonly found in wastewater are fibers, granules, fragments, and films, with the highest abundance of 91.32%, 70.38%, 65.43% and 21.36%, respectively [35]. MPs in the fiber-shaped environment originate from the washing and use of textiles; various cosmetics are the main sources of granules and fragments, while plastic films are formed from product packaging bags [39]. The shape of the MPs depends on the type of plastic being degraded, the residence time in the environment, and the type of degradation process. Studies have shown that the longer the residence time of MPs in the environment, the smoother and more rounded the edge becomes [40]. Differences in the size and shape of MPs make it difficult to remove them from the environment [41].

Due to their small size, MPs can be easily ingested by aquatic animals such as fish, birds, and other marine organisms, causing ecosystem disruption [42,43,44]. Many studies have shown the risk of human exposure to MPs, as they can enter the food chain, through contaminated waters, soils, and plants. Once ingested, they cause gastrointestinal obstruction and can bioaccumulate in organisms, causing negative health consequences, including obesity, asthma, respiratory irritation, cardiovascular disease, and cancer [45,46]. Some of the additives added to plastics are toxic and, when released from MPs, can penetrate cell membranes and interfere with biochemical reactions taking place in the cell, causing health problems [47].

MPs can adsorb contaminants present in environmental media due to their small size, high specific surface area, or lipophilicity, whereby the smaller the MPs and NPs, the greater the adsorption [48]. Atmospheric conditions, sunlight, pH, long exposure time, and hydrophobicity of persistent organic pollutants can significantly affect the adsorption kinetics of contaminants on MPs [49]. Studies have shown that MPs mainly adsorb heavy metals, pharmaceuticals, and personal care products, polycyclic aromatic hydrocarbons (PAHs), polychlorinated biphenyls (PCBs), per-/polyfluoroalkyl substances (PFASs), pesticides, and other organic pollutants [50,51,52,53,54,55,56].

To date, only a few papers have described the application of membrane techniques to MPs/NPs removal. In this review, the literature in this field has been reviewed to highlight the growing interest in the issue of plastic pollution and to demonstrate the still insufficient knowledge and experience in the removal of MPs and NPs, with a particular focus on the application of membrane technologies. This review analyzed around 200 articles published in the last 10 years with keywords including water treatment, microplastics, water and wastewater, and membrane processes. The articles were sourced from journal databases, namely Scopus and Web of Science. These articles were then discussed according to criteria including (1) efficiency of MPs/NPs removal in processes used in DWTP/WWTP, (2) efficiency of MPs/NPs removal by MF, UF, NF, RO, and MBR methods, and (3) effect of MPs/NPs on membrane fouling. The advantages of removing MPs from water/wastewater using membrane technologies compared to other removal methods are also discussed. In addition, studies on the release of MPs into water/wastewater from polymeric membranes are discussed.

## 2. Overview of Technologies for the Removal of Microplastics from the Aquatic Environment

**Figure 1:**
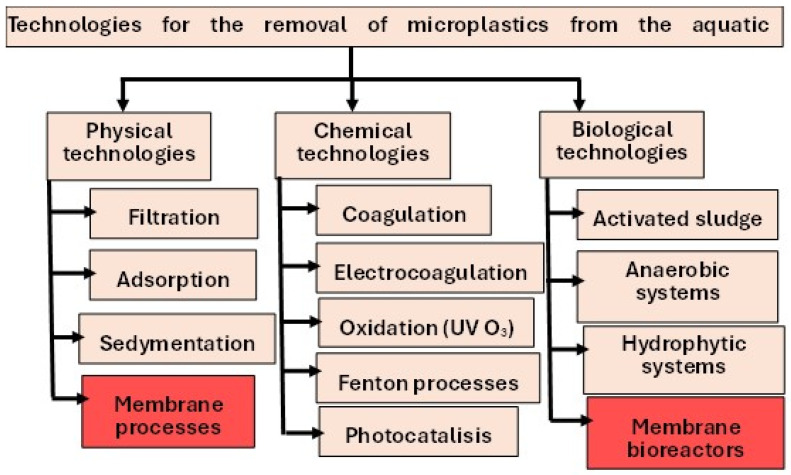
Overview of technologies for removing MPs from the aquatic environment. Own elaboration based on references [57,59,60,61].

In recent years, many technologies have been developed to remove MPs/NPs from the aquatic environment, which can be divided into physical, chemical, and biological, according to the principles of capture and removal (Figure 1) [57,58,59,60,61]. Based on the available literature, physical methods are studied more often than chemical and biological methods. Physical methods are mainly filtration, adsorption, sedimentation, flotation, and others, most of which have been tested in the laboratory and on a pilot scale, and some have even been implemented on a full scale. Among the various physical methods with high removal efficiency of MPs and NPs are carbon-based adsorbents such as biocarbon, magnetic carbon nanotubes, and magnetic adsorbents; disk and sand filters; dissolved air flotation; and magnetic separation processes [59,60,61]. Chemical methods are used in the treatment of water and wastewater containing MPs, alone or in combination, to enhance the effectiveness of physical processes (e.g., sedimentation, membrane processes). Among the chemical methods for the removal/degradation of plastics, ozonation, advanced oxidation processes, coagulation and electrocoagulation, Fenton processes, and photocatalysis are most commonly mentioned [60]. Biological methods use microorganisms to degrade and remove MPs present in the environment. Many organisms have been studied for their ability to degrade MPs in water and wastewater. The greatest ability to degrade MPs is demonstrated primarily by the activated sludge method, membrane bioreactors, and biological ponds and beds [60]. Biological methods of MPs removal have been used primarily for wastewater treatment, both municipal and industrial.

Some MPs in the influent to conventional DWTPs and WWTPs are removed from the water/wastewater by passing through a number of unit processes used at these plants. However, MPs are still present in both treated effluent from the WWTP and drinking water from the DWTP [10,12,40,62,63]. This requires further treatment to prevent re-introduction of MPs/NPs into the environment. For example, sludge from wastewater treatment plants containing MPs, if applied as fertilizer to agricultural fields, can cause contamination of agricultural soil [64]. On the other hand, processes such as photocatalysis and biodegradation treatments can degrade MPs into simple and harmless substances and even completely mineralize the polymers [65,66]. Although most DWTPs/WWTPs do not list MPs as target pollutants, more than 90% of MPs can be removed after a number of water and wastewater treatment processes [62,67,68]. However, due to the huge volume of treated water, a large number of MPs are still discharged into the environment by the DWTP/WWTP.

### 2.1. Wastewater Treatment Plants (WWTP)

Wastewater and solid waste are the main sources of MPs and NPs in the environment [69]. Many studies have confirmed their presence in saline, brackish, and fresh waters. The WWTP serving 650,000 people releases about 65 million MPs per day into natural waters [70]. In China, for example, more than 80% of MPs enter surface waters from the WWTP each year [71]. In addition, the concentration of MPs in sludge samples from 28 WWTPs ranged from 1.60 × 10^3^ to 56.4 × 10^3^ particles per kilogram of dry sludge [72]. The efficiency of conventional wastewater treatment in terms of MPs removal depends on a number of parameters. It is estimated that the efficiency of MPs removal in different regions of the world ranges from 60% to 90% depending on the wastewater treatment techniques used [73], with this value increasing to more than 97% with tertiary treatment [70]. The removal of MPs in WWTPs is therefore insufficient to prevent their release into the environment. It should be emphasized that MPs below 20 µm and NPs are not removed, which is why WWTPs are considered one of the main responsible for plastic pollution in natural waters [33,70,74]. Wastewater treatment can be divided into three main stages: pretreatment, biological (second stage) treatment, and tertiary treatment, also known as final or advanced treatment [75]. These stages include different methods, the use of which depends on the type of wastewater to be treated, as well as the assumed quality of the treated wastewater (Figure 1).

Pre-treatment starts with coarse screens to remove large floating elements, and the leachate is directed to the sand separator, which is basically a long, narrow tank that slows down the water flow and allows solids such as sand and gravel to settle out [76]. The effluent then flows to the primary settling tanks, where the suspended solids are deposited by gravity, forming the primary sludge. Coagulants and flocculants are sometimes added to facilitate the settling of suspended solids [77]. Pre-treatment generally involves the removal of large, suspended solids, but the liquid from this process still contains a large amount of suspended solids, and the removal efficiency of MPs is about 25%. It is also often used [78], which uses air bubbles to increase the buoyancy of contaminants on the water surface. At the WWTPs located in Hameenlinna in southern Finland, the removal rate of MPs that dissolved air flotation has been reported to be 95% [78]. In contrast to sedimentation, flotation can control the removal of low-density MPs (e.g., PE, PP, synthetic rubber (SR), and pieces of moderate-density plastics (e.g., PS and PA), which are difficult to separate by sedimentation [78].

Pollutants that have not been removed by pretreatment methods will be captured in one of the secondary treatment methods, which most commonly use a biological pollutant degradation process [60]. Activated sludge (AS) is a commonly used biological treatment consisting of an aeration tank and a secondary settling tank. The air provides oxygen to promote the growth of microorganisms that remove dissolved organic matter and reduce the biochemical oxygen demand (BOD) of the wastewater [79]. The wastewater then flows into a secondary settling tank, which allows sedimentation of activated sludge. Approximately 30% of the settled sludge is circulated, while the remaining secondary sludge is designed for treatment and disposal [79]. Various studies have shown that secondary treatment reduces MPs by 0.2–14% [70,80]. The main issue with the activated sludge process is the increasing accumulation of MPs by sludge flocs or bacterial extracellular polymers in the aerobic tank, and microorganisms can even degrade plastic particles present in the sludge flocs [81]. The efficiency of MPs removal during the activated sludge process is influenced by several factors, which include retention time and nutrient concentration in the wastewater [74,82]. For example, increasing retention time and nutrient concentration can significantly reduce the efficiency of MPs removal. In addition, a reduction in MPs removal efficiency can occur by increasing the reagent dose, inhibiting the rate of nitrogen conversion, and fouling the membrane in wastewater treatment processes [83].

A variant of the activated sludge process is the membrane bioreactor, in which an MF or UF module replaces the secondary settling tank used in the conventional activated sludge process [84]. Another variant of the activated sludge process is the anaerobic-anoxic-oxygen (A^2^O) method, whose main purpose is to remove nitrogen and phosphorus from wastewater. It consists of three tanks, namely an anaerobic tank, an anoxic tank, and an aerobic tank [85]. In the anaerobic tank, phosphorus is removed by microorganisms, while in the anoxic tank, nitrates are converted to gaseous nitrogen by denitrification. In the aerobic tank, BOD is removed, and nitrification takes place with the generation of nitrates through ammonia oxidation [86]. Effluent from the aerobic tank is returned to the anaerobic tank for further nitrate removal, while activated sludge from subsequent sedimentation is returned to the anaerobic tank for recycling of microorganisms [85].

Coagulation is sometimes used in second-stage treatment, which can have a positive effect on removal efficiency [70,87]. It was found that by using coagulation in the activated sludge process, the removal efficiency of MPs was increased from 54.4% to 66.7% [35,88,89]. During second-stage treatment, mainly MPs in the form of fragments are removed, while the sludge contains more fibers than other shapes of MPs [36]. It was also found that MPs larger than 500 μm are eliminated during second-stage treatment, while smaller fragments are usually present in the treated effluent [90].

Increasingly, tertiary treatment is being used to further remove pollutants from wastewater after the secondary treatment stage, allowing for a higher quality of treated wastewater [91]. Wastewater tertiary treatment mainly uses processes/technologies with high MPs/NPs removal efficiency, and these include chlorination, ozonation, UV radiation, disk filter, rapid sand filtration, granular activated carbon, photocatalysis, and others [91]. In tertiary treatment, MPs/NPs removal efficiencies of 98% can be achieved, which sometimes corresponds to a quality close to drinking water [70,74,92,93].

The removal efficiency of MPs at different treatment stages in WWTPs, which has been described by Sun et al., 2019 [94], is summarized in Table 2. However, despite the high level of plastic removal, a huge amount of treated wastewater is considered to be a source of plastics in wastewater [33].

Among tertiary treatment processes, membrane operations can be an effective efficient solution to the problem of MPs and NPs contamination in wastewater. The application of membrane technology for the treatment of wastewater contaminated with MPs is of increasing interest due to the wide range of membrane properties, such as large surface area, porosity, and others.

### 2.2. Drinking Water Treatment Plants (DWTP)

Water treatment is the process by which water from surface or underground sources is brought to the state of purity required for the application. Water can be treated for social and domestic as well as industrial purposes. Different methods of drinking water treatment are used [95]. The technological system is selected based on a detailed analysis of the water composition. Drinking water treatment processes include coagulation, sedimentation, aeration, mechanical filtration, activated carbon filtration, softening, ozonation, and disinfection—UV lamp irradiation and chlorination [95]. Disinfection is the final step to protect the water from the growth of bacteria, viruses, and other pathogenic microorganisms before it reaches the consumer. Most of the research on MPs removal in the DWTPs system has focused on particular water treatment technologies.

Ma et al. [96,97] evaluated the effectiveness of coagulation/flocculation in removing PE during drinking water treatment. They found that aluminum coagulant (AlCl_3_·6H_2_O) was more effective than iron coagulant (FeCl_3_·6H_2_O) in removing PE, and that the removal efficiency increased with decreasing PE particle size from 5000 to 500 µm at a constant coagulant dose. Increasing the dose of AlCl_3_·6H_2_O only slightly increased the removal efficiency, especially of larger PE particles. It should be noted that the effectiveness of coagulants in removing MPs depends on both concentration and pH. The addition of anionic polyacrylamide (PAM) as a flocculant significantly increases the removal of smaller PE particles (<500 µm) [96]. The highest removal efficiency of MPs by coagulation was 61.2%.

A study by Pivokonsky et al. [98] for three DWTPs in the Czech Republic showed the average removal efficiency of MPs particles from >100 µm to 1 µm was between 70 and 83% during water treatment, including coagulation/flocculation, sedimentation or flotation, and filtration with sand and activated carbon. Unfortunately, the treated water contained as many as 628 MPs per liter, with 95% being less than 10 µm.

Wang et al. [99] studied MPs removal in a DWTP station using coagulation/flocculation, sedimentation, sand filtration, ozonation and granular activated carbon filtration. The DWTP was found to have an overall MPs removal efficiency of 82.1–88.6%, with approximately 84.4–86.7% of MPs particles in the influent having sizes between 1 and 5 µm. Coagulation and sedimentation removed 40.5–54.5% of MPs from the raw water, which was comparable to the results obtained by Ma et al. [96] using AlCl_3_-6H_2_O and polyacrylamide as coagulant and flocculant.

Zhang et al. [100] investigated a coagulation-flocculation-sedimentation process using Al_2_(SO_4_)_3_ as a coagulant and polydiallyldimethylammonium chloride as a flocculant, obtaining MPs removal efficiencies of 58.9–70.5%, which is below the average removal efficiency obtained in other studies. Sand filtration removed 29.0–44.4% of MPs and was more effective for larger particles >5 µm. The use of ozonation in addition resulted in a slight increase in the amount of MPs removed, probably due to the additional removal of organic matter from the MPs particles. Ozonation and granular activated carbon filtration together provided an additional 17.2–22.2% MPs removal efficiency.

## 3. Membrane Techniques

### 3.1. Fundamentals of Pressure-Driven Membrane Techniques

**Figure 2:**
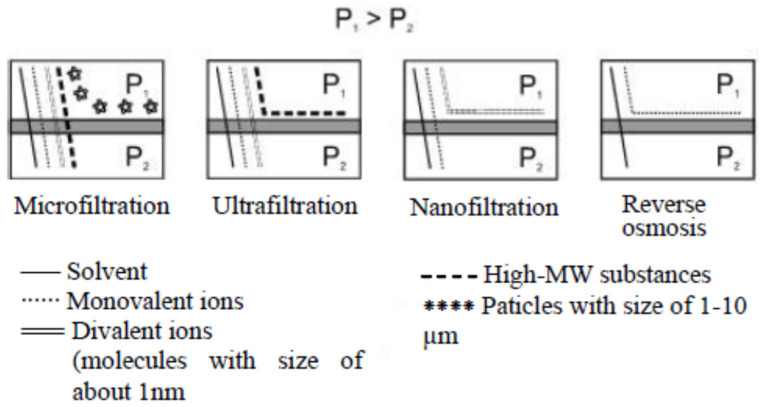
Differences in efficiency of pressure-driven membrane processes; own elaboration based on references [101,102,103].

Depending on the membrane pore size, the separation mechanism and the size of the particles/molecules to be separated, pressure-driven membrane techniques include: microfiltration (MF), ultrafiltration (UF), nanofiltration (NF), and reverse osmosis (RO) [101,102,103]. Table 3 summarizes the main features of these processes. Under applied pressure, solvent and low-molecular-weight (MW) solutes pass through the membrane, while other higher-MW molecules as well as colloids and fine suspensions are retained by the membrane. Depending on whether one is dealing with MF, UF, NF, or RO, particles with increasingly smaller MW are retained. The area of application determines the size of the particles retained by the membrane. Therefore, the hydraulic resistance of membranes is increasing and higher transmembrane pressures (TMPs) are being used. Taking the particle size of the substance retained by the membrane as a basis for classification, Figure 2 shows schematically the difference between the different membrane pressure-driven processes when separating suspensions and real solutions.

The membrane is the core of any membrane technique, and the other instrumentation only contributes to the optimal operation of the membrane. With respect to the material from which the membrane is made, a distinction is made between solid and liquid membranes and between organic (polymeric) and inorganic membranes, while with respect to the structure and morphology of membranes, they can be divided into porous and solid (non-porous), symmetric and asymmetric, and composite membranes, which are considered a variation in asymmetric membranes. Asymmetric membranes have a thin selective layer over which is a thicker layer with high permeability [101,102,103]. They are used in most membrane processes. Most commonly, semi-permeable membranes are made of synthetic organic polymers. These include polyethylene (PE), polytetrafluoroethylene (PTFE), polypropylene, and cellulose acetate (CA) [103]. Inorganic membranes, on the other hand, are made of materials such as ceramics, metals, zeolites, or silica. Non-organic membranes can be divided into ceramic membranes, stainless steel membranes (obtained by sintering stainless steel powders), carbon membranes (obtained from graphite pastes or carbon fabrics), and glass membranes [103]. The most important are ceramic membranes, whose materials are aluminocrystals and oxides of aluminum (α-(alumina) and γ-varieties), titanium, zirconium, and silicon.

The configurations of available membrane modules are based on two basic forms of membrane: flat or tubular, and they are available in five basic types: plate-frame modules, spiral modules, tubular modules, capillary modules, and hollow fiber modules. Plate-frame and spiral solutions are based on membranes in the form of flat sheets, while tubular, capillary, and hollow fiber solutions are based on membranes with a circular cross-section [103,104]. In the plate-and-frame module, two flat membranes, between which the feed flows, are arranged in parallel to each other so that the epidermal layers face the feed stream, separated by a spacer plate (e.g., plastic mesh) and a porous/waved support plate to guide the permeate, forming the basic assembly. The spiral module is a retrofit of the plate-and-frame module design, especially considering the packing density of the modules, which can be as high as approximately 1000 m^2^/m^3^. The flat membrane, together with a spacer plate, is wound spirally onto a central collection tube. The spacer plates on the feed and permeate sides are then welded on three sides to form an envelope/pocket for the membrane. Such an envelope thus consists of two diaphragms, with a spacer plate and permeate discharge plate between them. The feed solution flows axially through the cylindrical module, parallel to the central pipe, while the permeate flows radially into the central pipe. The principle of tubular module construction is to form a tube-shaped membrane and embed it inside or outside a porous or perforated support pipe. In the first solution, the feed solution usually flows inside the tube and the permeate, after passing through the membrane, flows in the porous support or exits the support through the perforation holes. In the second, on the other hand, the porous pipe slide is often covered with a membrane-forming layer, creating a compact membrane-pipe system. In a typical arrangement, tubes with a diameter of 10–25 mm are used. In order to increase the relatively low packing density, a number of such tubes are placed in a pressurized housing (shell), and the module then resembles a shell-and-tube heat exchanger in design. The capillary module consists of a large number of capillaries (bundle), 0.5–1 mm in diameter, placed in a cylindrical casing 0.8–1.0 m in diameter and approximately 1 m long and glued after sealing with epoxy resin, polyurethane, or silicone rubber. In this solution, the membranes (capillaries) are not supported as in the case of classical tubular modules. Hollow fiber modules (hollow fibers) are similar to capillary modules, but the outer diameter is much smaller, ranging from 80 to 200 (100) µm, and the wall thickness is in the order of 20 µm. However, larger fiber wall thicknesses are sometimes used so that they can withstand large pressure differentials of up to 8 MPa.

Membrane material, pore size, thickness, and surface characteristics affect the performance of the membrane process. The main disadvantage of membrane filtration is the phenomenon of membrane fouling, which occurs as a result of the adsorption of particles on the surface and inside the membrane pores. As a consequence of membrane fouling, the performance of membrane processes is reduced, resulting in higher energy consumption, increased operating time, and maintenance costs [101,102,103,104,105]. Enfrin et al. [105] showed that MPs can interact with the membrane surface due to their intrinsic physicochemical properties, such as hydrophobicity, surface charge, and roughness. Nevertheless, membrane technology is highly effective in removing low-molecular-weight contaminants and MPs of different sizes and shapes and NPs. Considering the advantages and possibilities of using membrane techniques, they are considered a green technology [106,107].

### 3.2. Membrane Methods for Removal of MPs and NPs

Membrane filtration is increasingly used in the initial, intermediate, and final stages of wastewater treatment and water treatment, as well as in domestic filtration equipment [108]. The overall removal efficiency of MPs/NPs in conventional DWTPs and WWTPs increases after the introduction of membrane technologies [62,109]. This is due to the fact that membranes have smaller pore sizes not only than MPs but also than some NPs, making their removal efficiency superior to other technologies used in tertiary wastewater treatment and advanced drinking water treatment.

#### 3.2.1. Micro- and Ultrafiltration

The MF process uses membranes with pore sizes between 0.1 and 10 μm and a TMP range of 0 to 2 bar, while UF membranes have much smaller pore sizes in the range of 0.1 to 100 nm [101,102,106]. If we compare these sizes with the particle size of MPs (5 mm–1 µm) and NPs (<1 µm), it can be assumed that MF and UF membranes should retain MPs, while UF should also retain NPs depending on the pore size of the membrane. Polymeric membranes are characterized by swelling, biofouling, and poor thermal and chemical resistance, leading to a short service life and requiring regular cleaning procedures. In addition, polymeric membranes can release polymer particles, thus adding MPs to the permeate. Ceramic membranes (e.g., SiC, ZrO_2_, and Al_2_O_3_), on the other hand, have higher chemical and physical stability, longer service life and lower maintenance costs, leading to a significant reduction in operating costs and environmental impact. Therefore, ceramic membranes are much more advantageous to use than polymeric membranes, especially for MF/UF of industrial wastewater with high temperature, difficult chemical conditions, and containing abrasive particles [110]. Titanium oxide, alumina, and other materials have been used to prepare ceramic MF/UF membranes for MPs removal [111].

In studies of the removal of MPs from wastewater after biological treatment by MF, using a submerged MF membrane with a pore size of 0.1 μm, a 98–100% reduction in MPs concentration was achieved, from 81 to 106 MPs/L to only 1–2 MPs/L [111]. The particles detected in the permeate were smaller than 0.1 μm, indicating that they were NPs.

The UF process used to remove MPs simultaneously allows for the removal of other contaminants present in water and wastewater, such as macromolecular compounds, bacteria, protozoa, viruses, and suspended solids. UF is often used in combination with other methods as a second treatment step for wastewater (membrane bioreactors) or water (in combination with coagulation) [6]. As already mentioned, the particle size of MPs is larger than the pores of the UF membrane, making them completely retained by UF membranes [97]. During UF MPs, there is a 38% decrease in the final water flux as a result of the interaction between MPs and the membrane surface and pores, i.e., adsorption on the surface and inside the membrane pores [112]. In contrast, Ma et al. [96] obtained as much as a 66% decrease in permeate flux due to fouling caused by PE MPs, with removal efficiencies of <91% and particle size <0.5 mm.

Pizzichetti et al. [113] presented an evaluation of the performance and removal efficiency of MPs from PA and PS of 20–300 μm by UF membranes of PA, CA, and PTFE with a pore size of 5 μm. MPs removal efficiencies above 94% were obtained for all three membranes, with cellulose acetate being the most effective membrane material (Table 4). For comparable overall mass removal efficiencies, the optimal membrane is that operating with the lower TMP, and therefore the lower pumping costs. PTFE, due to its hydrophobicity, requires a high working pressure, negatively affecting the pumping costs. PC and CA membranes have similar behavior during PA filtration. However, during PS filtration, CA allowed higher water flux. The main mechanism was sieve separation by particle size, but membrane abrasion and fouling phenomena caused MPs particles to either pass through the membrane or fragment into smaller particles, depending on membrane properties, MPs–membrane interaction, particle size, and TMPs used.

The paper in reference [114] presents the results of a study on the removal of MPs from industrial wastewater by UF using a polyacrylonitrile (PAN) composite membrane with reduced graphene oxide (rGO/PAN). Studies have shown that higher amounts of rGO (0.11 to 0.83 wt.%) added to the PAN matrix result in a larger number of pores of similar size (~150 nm), allowing the separation of colloids (>82%) and, more importantly, MPs. Important features of the rGO/PAN composite membranes tested are their anti-fouling properties and the ease of cleaning the filter cake layer, which allows them to be reused. Furthermore, it has been shown that multi-stage treatment of wastewater containing MPs can be replaced by a single membrane process using rGO/PAN composite membranes. Yang et al. [115] investigated the fate and distribution of MPs and the effectiveness of UF in several natural water sources and wastewater treatment plants (seawater, municipal wastewater, pharmaceutical factory wastewater, and drinking water treatment plants) in France. The 200 kDa UF membrane resulted in 70–100% and 80–100% removal of MPs from PE in DWTPs and WWTPs, respectively. In addition, the UF process allowed MPs to control size in the ranges below and above 150 μm. Gonzalez et al. [116] compared the removal of MPs from municipal wastewater and plastic industry wastewater using UF and rapid gravity filtration. They found that rapid gravity filtration did not result in a corresponding reduction in MPs concentration (about 40%), whereas. UF successfully removed MPs from wastewater at virtually 100% MPs.

Table 5 shows the retention rates of PS and bovine serum albumin (BSA) nanospheres obtained with both UF and MF membranes [117]. The pore size of the membranes studied determined the membrane separation efficiency due to the steric exclusion of the compounds studied. In this study, an MF membrane made of chlorinated PE (pore size: 0.4 µm) and two UF membranes (made of regenerated cellulose (RC) and polyethersulfone (PES) with a cut-off of 30 kDa) were used to remove single and mixed solutions of nanospheres from PS (120 and 500 nm) and BSA (66 kDa) [117]. As expected, both UF membranes showed high retention rates of NPs from PS and BSA due to the molecular sieving mechanism. However, electrostatic interactions have also been demonstrated to play a key role in the separation mechanism of compounds such as proteins by UF membranes. In this context, the RC membrane studied was slightly negative for pH > 3, with a zeta-potential value near −2 mV at pH 7, whilst the PES membrane had a much higher negative zeta-potential value (−15.7 mV). Considering that the BSA was also negatively charged, the electrostatic repulsion forces between the PES membrane and BSA were much higher than the RC and BSA. Consequently, the BSA rejection coefficient obtained with the PES membrane was higher than with the RC membrane.

For the removal of MPs/NPs from laundry wastewater, MF and UF processes have been proposed. A study by Luogo et al. [110] compared the performance of a silicon carbide (SiC) MF membrane and a zirconium oxide (ZrO_2_) UF membrane in the treatment of laundry wastewater. The filtration of the synthetic feed with nylon fibers of 80 μm showed a critical flux value, in the case of MF, of 200 L/(m^2^h). This is an effect of MPs in terms of pore blocking, but for the UF membrane, it was not possible to obtain a critical flux because no reduction in flux was observed along with the increasing TMP cycles. This means that the fouling occurs earlier in MF compared to UF. In both cases, a 100% rate of removal of the fibers was achieved. For the filtration of the real wastewater from the tent laundry outlet, the critical flux value, and backflush period for the MF was 90 L/(m^2^h) with a 20 min period and 50 L/(m^2^h) and 60 min period for the UF. After 4 days of constant filtration, there was a considerable decrease in the permeability of MF (~95%), while much smaller in the case of UF (~37%). The obtained MPs removal efficiencies from the wash effluent in terms of total solids, turbidity, and MPs concentration were higher for UF than for MF, at 99.2% and 98.55%, respectively.

In many cases, MF/UF are integrated with classical technologies used in water and wastewater treatment, such as sedimentation, classical filtration, flotation, biological, and advanced oxidation processes [96,97]. UF integrated with the coagulation is very frequently used in water treatment plants, thanks to high removal of organic matter. Several studies have analyzed the removal and impact of MPs by membrane processes in laboratory-scale DWTPs [96,97,118,119], using UF, MF or RO, while a small number of studies have investigated the removal of MPs by membranes in full-scale DWTPs [62,120]. MPs in the studies conducted were generally larger than the pore sizes of the membranes, resulting in virtually complete removal of MPs in all studies.

If high concentrations of MPs are present in natural freshwaters, in-depth studies of this process should be conducted, due to the fact that it is a technology used for drinking water production [121,122,123]. A study by Ma et al. [96,97] investigated the removal of PE in drinking water treatment using UF on a polyvinyl membrane and coagulation with the coagulants FeCl_3_∙6H_2_O and AlCl_3_∙6H_2_O. The density of PE is 0.92–0.97 g/cm^3^ and is very close to that of water, making it difficult to remove by sedimentation or even flotation. In the conventional treatment system, the overall PE removal efficiency was 86.14%, while after UF, PE particles were removed to a much higher degree due to the small pore diameter of the UF membrane. It was found that there was a slight fouling of the membrane after coagulation at the conventional coagulant dose, especially for large PE particles [96,97]. As the coagulant dose increased, the fouling gradually increased due to the formation of a thicker precipitate layer. In order to increase the efficiency of the coagulation process, a flocculant in the form of polyacrylamide (PAM) was used in this study, which increased the removal efficiency of smaller MPs and NPs, improved the yield, and reduced fouling due to the opposite charge of PAM to that of the coagulant flocs [96,97].

The main mechanism of MPs removal by MF and UF membranes is the sieve mechanism (exclusion by size), i.e., theoretically, MPs larger than the membrane pore size are retained by the membrane, and smaller ones pass through the membrane [10]. In addition, adsorption of MPs on the membrane surface and intra-pore hydrophobic interactions and electrostatic repulsion forces are further mechanisms for MPs removal by MF and UF membranes. In particular, the hydrophilicity and zeta potential of MPs and membrane influence the interaction forces between the membrane surface and MPs [121].Thus, the characteristics of MPs particles (such as size, shape, and polymer type) and membranes (such as structure, pore size, and membrane material) determine the removal of MPs.

In conclusion, it should be emphasized that recently there has been an increasing amount of research into the use of MF and UF membranes for MPs remediation. However, one of the research gaps, in this field, is the scarce knowledge of the removal efficiency of MPs of different sizes, shapes, and chemical compositions. Most studies focus on the removal of MPs in the 1–100 μm range, while there is a lack of information on the removal efficiency of smaller or larger MPs. Furthermore, there is a need to investigate the long-term stability of membrane performance and efficiency in the presence of MPs, as well as the fouling and degradation potential of MPs. Methods to minimize the presence of MPs in water filtered through UF membranes are not known, as MPs particles that are smaller than the membrane pores may still pass through the membrane pores. Further research is needed to optimize and model the operation of MF/UF membrane systems for MPs remediation, with a focus on increasing efficiency, reducing energy consumption, and minimizing environmental impact.

#### 3.2.2. Nanofiltration

Nanofiltration (NF) is a pressure-driven membrane technique by the difference with properties intermediate between RO and UF (Figure 2). Membranes for NF are characterized by low retention of monovalent ions and high retention of bivalent ions and organic compounds with molecular weights above 200–300 Da [101,102,103,104,105]. In NF, solution components with particle sizes of about 1–3 nm are separated and the pressure difference lies in the range of 1–3 MPa, which is below the value that would be necessary in RO to obtain the same fluxes. The membranes used in NF do not have pores in the conventional sense. The mechanism of separation of the solutes from the solvent is, therefore, by dissolution and diffusion in the membrane. The permeation of salts is determined by the valence of the ion, with retention rates of cations and anions increasing in the order of

NO_3_^−^ < Cl^−^ < OH^−^ < SO_4_^2−^ < CO_3_^2−^,

H^+^ < Na^+^ < K^+^ < Ca^2+^ < Mg^2+^ < Cu^2+^.

In contrast to membranes for RO, electrical effects occur during NF of ionic solutions, determined by the fixed charges, mainly negative (−COOH or −SO_3_H), of the membrane surface or its pores. Due to electrostatic interactions, the membrane charge interferes with the permeation of multivalent ions, resulting in the possibility of fractionation of monovalent and divalent ions. If NF membranes are used to desalinate solutions containing monovalent and multivalent ions, there is an effect, called the Donnan effect, whereby the retention factor of the Cl^−^ ion takes on negative values as the concentration of Na_2_SO_4_ increases. This means that the chloride anion is transported against its own concentration gradient to maintain equilibrium between the ion charges in the two phases. To date, NF has been successfully applied on a technical scale in groundwater and surface water treatment processes, primarily for softening and for substance separation in industrial processes.

As a typical representative of membrane technology, NF is considered a promising method for advanced wastewater treatment after the second stage, as it can effectively remove inorganic salts, organic matter, bacteria, and large solids, such as MPs [124].

Air pollution caused by various hazards such as particulate matter, MPs, bioaerosols, etc., has become a global public health concern worldwide. In recent decades, NF-based air cleaning techniques have rapidly evolved as a viable solution to address air pollution challenges worldwide [125]. The developed NF membrane has an excellent capture efficiency of over 99% for PM0.3, MPs0.3, and BA.

Studies have also been conducted to determine the effectiveness of MPs removal from landfills [126]. The effluent from the UF and NF units had MPs concentrations of 7.24 particles/L and 2 particles/L, respectively. The results showed that the NF process was 99% effective in removing MPs; for UF, about 96% MPs removal was achieved. Furthermore, it was found that the most common type of MPs in all studies was fiber, with the main sizes ranging from 500 to 999 μm and 1000 to 1999 μm. The differences in efficiency were due to differences in pore size of NF and UF membranes.

Severino et al. [127] investigated an integrated process that combines membrane separation with a photocatalytic process to remove NPs from water. The NF process achieved 100% retention of NPs, increasing their concentration from 2 mg/L to 100 mg/L in the concentrate and reducing the effluent volume with a volume reduction factor of 44.25. In addition, little effect on membrane fouling was observed, with almost complete restoration of the initial membrane performance (98%) after water washing. The next step was photocatalytic degradation of concentrated wastewater using solid-state titanium dioxide (TiO_2_) photocatalysts. The results showed the mineralization of 10 mg/L PS NPs after 24 h, using 1 g/L TiO_2_ under UV light This study demonstrates a promising system that combines recovery and degradation in a single optimized step, paving the way for application to the third stage of wastewater treatment in wastewater treatment plants (WWTP).

A study was also carried out to assess MPs contamination in river and drinking water inflows at a drinking water treatment plant (DWTP) in the Paris region of France, which used the NF process [128]. The drinking water distribution network was also investigated by sampling at three points in the network. Concentrations of MPs ranging from 7.4 to 45.0 MP/L were found in the inlet water, while concentrations ranging from blank levels (0.003 MP/L) to 0.260 MP/L were in the outlet drinking water (overall removal rate above 99%). PE, PP, and PET were the main polymers found at both the inlet and outlet, but the proportions differed significantly at the outlet. Concentrations in the distribution network were generally higher than at the corresponding DWTP outlet, although a high degree of inter-sample variability was observed. Our results suggest that membrane processes such as NF are more efficient than typical purification processes and that re-contamination of MPs in the distribution network itself may occur.

#### 3.2.3. Reverse Osmosis

Reverse osmosis (RO) is a pressure-driven membrane process in which TMP induces the selective movement of solvent molecules in the opposite direction from the osmotic pressure, i.e., from a solution with high osmotic pressure to a solution with low osmotic pressure (water). In the RO process, low-molecular substances (e.g., monovalent salts, undissociated acids, organic compounds) are retained. The separation mechanism in the RO process is described by the dissolution-diffusion model, which assumes that the flow of specific components through compact polymer membranes is determined by their dissolution in the polymer and diffusion. TMPs used in the RO process must exceed the osmotic pressure of the feed and are generally in the range of 1.0–8.0 MPa [129]. RO is the basis of one method for desalination of saline and brackish waters. It is also used for the treatment and concentration of industrial wastewater from various industries and for the recovery of water and other substances contained in the wastewater [96,97,130]. The main advantage of RO is the relatively low energy consumption compared to thermal methods of water desalination, as the process occurs without phase transformation.

RO is used for the removal of MPs and NPs primarily as a third or even fourth stage of wastewater treatment with efficiencies exceeding 90% [40,107,131]. However, studies in recent years have shown that treated wastewater can contain significant amounts of MPs, even after passing through RO membranes used as a third treatment step [40,109,132]. Cai et al. [107] reported that MPs in the effluent of WWTPs using pre-sedimentation, biological treatment, and MBR and RO processes achieved 93.2% and 98.0% MPs removal efficiency, respectively. Furthermore, they found that non-fibrous MPs larger than 0.5 mm were completely removed from the effluent, while MPs with a fiber structure, especially those of 200 μm, passed through RO membranes into the treated effluent [110].

The pore sizes of RO membranes are small enough to retain not only MPs but also NPs with high efficiency (permeate flux) [7]. Nevertheless, NPs fibers have been found in water samples after RO treatment [133]. New research in relation to NPs removal has shown that the combination of UF and RO processes guarantees consistent performance in terms of permeate flux and quality [134]. Thus, the use of RO membranes after membranes with larger pore sizes and higher cut-off (UF/MF) in WWTPs contributes to the presence of fewer MPs and NPs in the effluent leaving the treatment plant. This arrangement allows almost complete removal of NPs, except for those that pass through membrane defects, plant leaks [40], or worn polymer membranes [132]. Therefore, there is a need to clarify to what extent MPs/NPs after treatment with polymeric membranes can originate from the membrane material and to take measures to prevent this phenomenon. The main disadvantages of RO technology are the relatively high energy requirement, membrane fouling, and concentrate (retentate) management [135].

Ziajahromi et al. [40] studied the removal efficiency of MPs and NPs at a WWTP in Sydney, Australia. The treatment plant produces highly treated effluent through the use of first-, second-, and third-stage treatment processes, which include screening and sedimentation, biological treatment, flocculation, disinfection/dechlorination processes, UF, RO, and decarbonization, and the treated effluent is discharged to the river. After the first, second, and third treatment stages, MPs/NPs were still present in the leachate [40]. In particular, MPs/NPs with an irregular shape were detected and identified by Fourier transform infrared spectroscopy. In addition, MPs fibers were found to be present in the samples after the RO process, with a removal efficiency of 90.45% for MPs > 25 µm [40], with fibers accounting for 88% of all MPs. After four treatment stages, comprising first-, second-, and third-stage treatment processes and RO, the wastewater treatment plant continues to release ten million plastic wastes per day into the natural aquatic environment [40].

**Figure 3:**
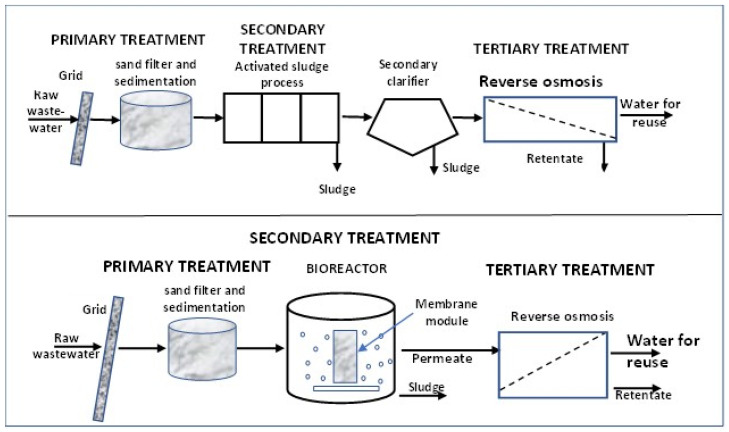
Concepts for the application of the RO process in wastewater treatment; own elaboration based on references [40,109].

The paper [109] presents the results of MPs removal in an integrated membrane system (IMS) and classical activated sludge treatment. The classical system included grids, a sand filter, a sedimentation tank, activated sludge, and a secondary settling tank, while IMS pretreatment and MBR, UF, and RO (Figure 3). The MBR was equipped with capillary PVDF membranes with a pore size of 0.4 μm, operating at a capacity of 1.50 × 10^8^ m^3^/d, while the RO was constructed with flat membranes with a pore size of 0.0001 μm and a capacity of 4.0 × 10^7^ m^3^/d. The water recovered by the RO process could be reused as industrial water. The removal of MPs in the IMS after treatment in the MBR was 93.2% and increased to 98.0% after RO. The concentration of MPs in the MBR leachate was reduced from 1.5 × 10^13^ MPs/d to 10.2 × 10^11^ MPs/d, and in the RO process to 2.7 × 10^11^ MPs/d [109]. Membrane treatment included MPs of different types, sizes, and shapes. The results showed that IMS was more effective in removing MPs from wastewater, but the possibility that fine fibers (<200 μm) could pass through an IMS, even equipped with RO.

Wang et al. [136] studied the removal of phthalate esters (dimethyl phthalate, dibutyl phthalate, di-isobutyl phthalate, and di(2-ethylhexyl) phthalate) and MPs from wastewater simultaneously in four treatment plants and tanks. Clarification, filtration, and RO were used, and the removal of phthalate esters and MPs in all treatment plants was 47.7–81.6% and 63.5–95.4%, respectively. MPs in the form of granules and fragments (<0.01 mm in size) were present in the effluent with concentrations of 276–1030 MPs/L and −103–4458 MPs/L in the receiving water bodies.

#### 3.2.4. Forward Osmosis

**Figure 4:**
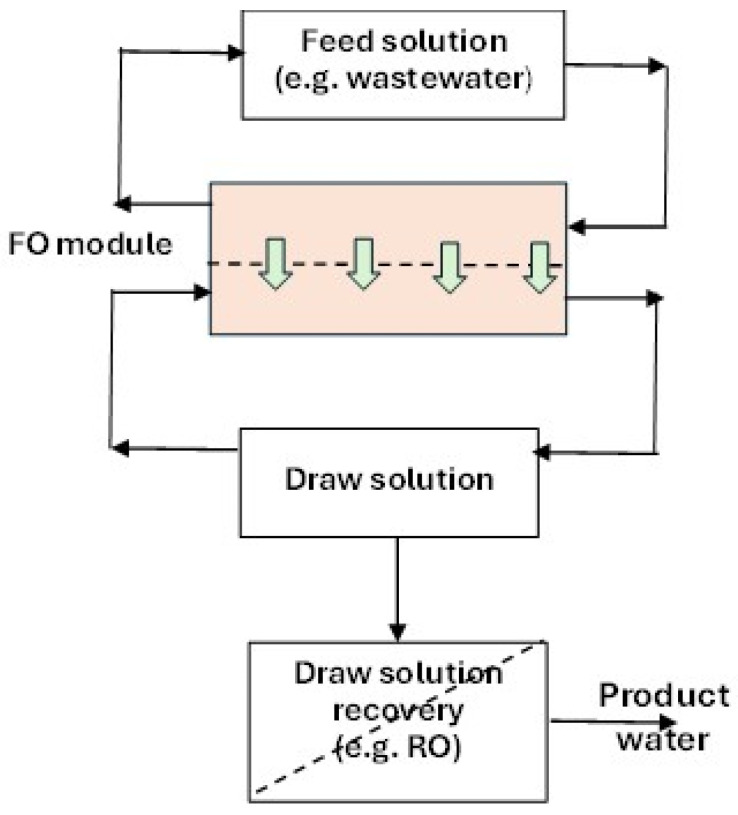
Schematic of forward osmosis (FO); own elaboration based on references [137,138].

Forward osmosis (FO) is a technological process that uses the phenomenon of osmosis, i.e., the diffusion of a solvent across a semipermeable membrane separating two solutions of different concentrations [137,138]. The FO process requires a solution with a high salt concentration (draw solution—DS) to recover water from a feed solution (FS), such as wastewater (Figure 4) [139,140]. The high concentration DS provides the concentration gradient on both sides of the membrane and thus the required osmotic pressure difference and causes the transport of water molecules from the FS to the DS until the chemical potential equilibrium is reached [141]. The driving force of the process is generated naturally and is the result of the difference in osmotic pressure of the solutions on the two sides of the membrane. The water flow is spontaneous and the process does not require any external energy (apart from the energy associated with the circulation of the solutions on the two sides of the membrane). The water that has permeated the membrane causes dilution of the DS and hence the next step is its regeneration, resulting in two streams—pure water and the recovered concentrated osmotic solution, which can be reused [137].

The performance of the FO process is determined by the properties of the DS, which should have high osmotic pressure at the lowest possible concentration, low viscosity, ease of recovery, low cost, and must not be toxic. In addition, the back diffusion of osmotic solution components into the FS should be limited. The following have been used as DS components: water-soluble gases (SO_2_ or a mixture of NH_3_ and CO_2_), sugars (glucose, fructose, sucrose), inorganic salts (NaCl, MgCl_2_, CaCl_2_, Al_2_(SO_4_)_3_), organic salts (sodium+ and magnesium salts of formic, acetic, or propionic acid), and hydrophilic magnetic nanoparticles [137,138]. In the FO process, as in RO, non-porous asymmetric membranes made of hydrophilic polymers, i.e., cellulose triacetate, or composite membranes containing a polyamide active layer are used [137,138]. Compared to pressure-driven membrane processes, the FO process has a number of advantages, including higher retention of contaminants from wastewater compared to MF and UF, high membrane ‘cut-off’, low operating pressure compared to NF and RO, and a relatively low propensity for membrane fouling [142].

In wastewater treatment, UF/NF and RO can be fully or partially replaced by FO systems, especially for difficult contaminants with a high propensity for fouling [143]. Studies have shown the high retention efficiency of trace contaminants in the FO process. Valladares Linares et al. [144] reported that the retention rate of thirteen trace contaminants, including five hydrophilic, three hydrophobic non-ionic, and four hydrophilic ionic contaminants, ranged from 67.9% to 98.9%. A study of the removal of 23 trace contaminants using the FO method showed that the retention of charged substances was above 80%, while neutral compounds ranged from 40% to 90% [145]. In contrast, Jin et al. [146] observed that the retention of pharmaceutical trace contaminants using CTA and thin-film composite FO membranes was 96% and 93%, respectively. FO was found to be more effective than other separation processes in removing toxic algae (~90%) [147], antibiotics (~87%) [148], micropollutants (~98%), and oxidation by-products (~89%) [149].

In the last decade, a fertilizer-driven FO (FDFO) process has been developed to recover water from wastewater, which can be directly used for irrigation of hydroponic systems [150]. Wang et al. [151] investigated the removal of MPs/NPs by the FDFO process using cellulose triacetate flat FO membranes. FS was prepared by adding different concentrations of isolated bacterial extracellular polymers (5 mL/L concentration) and model plastics (1 µm and 100 nm PS with 5 mg/L concentration) in deionized water, while KCl solution (1 mol/L) was used as DS. The FS and DS solutions were separately circulated on both sides of the FO membranes, and no MPs and NPs were detected in the water after the FDFO process. The results of the experiment proved that the FDFO process can generate high-quality water from wastewater by eliminating all contaminants present in the wastewater. Good removal of MPs and NPs from wastewater can significantly reduce the risk of contaminant-related plastics entering the environment. As with other membrane filtration processes, membrane fouling is also a major challenge to the stability of the FDFO process [152].

#### 3.2.5. Membrane Bioreactors

**Figure 5:**
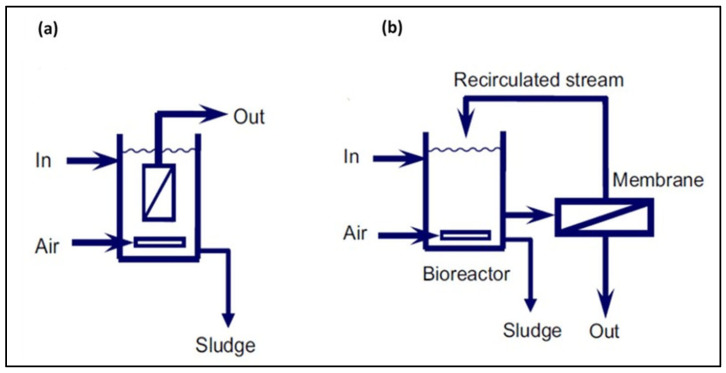
Membrane bioreactor (MBR) configurations: (**a**) membrane module immersed in the bioreactor and (**b**) membrane module outside the bioreactor; own elaboration based on references [7,153].

**Figure 6:**
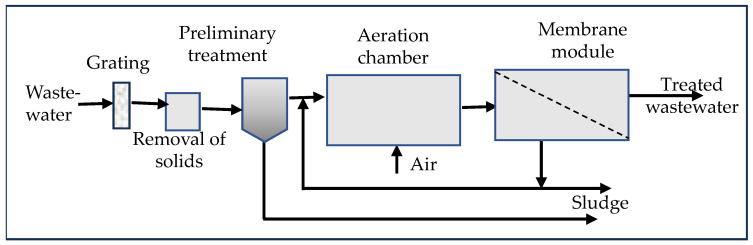
Schematic of wastewater treatment using MBR; own elaboration based on references [7,153].

Membrane bioreactors (MBRs) are systems in which a process supported by biological catalysts (bacteria, enzymes) is combined with a membrane process [7,153]. The membrane module (MF or UF) can be installed outside the bioreactor or immersed in the bioreactor (Figure 5) [7,153]. By using membrane filtration, the secondary settling tank used in a classical activated sludge system can be eliminated, avoiding the loss of biomass in the effluent and achieving a high biomass concentration (Figure 6) [4,154].

Membranes used in MBR should be characterized by durable hydrophilicity, excellent oxidation resistance, high mechanical strength, and provide high filtrate flux and filtration accuracy. MBR fits perfectly into the principles of green chemistry, following the logic of process intensification, which offers new and much greater opportunities for competitiveness, improved product quality and novelty, process innovation, and environmental friendliness [155]. Today, MBR is considered one of the most efficient technologies for the effective treatment of municipal and industrial wastewater [7,155], due to the high quality of treated wastewater, the small footprint, the complete separation of hydraulic retention time (HRT) and sludge retention time (SRT), and the ease of scaling up.

Combining pressure-driven membrane techniques with a biological process (MBR) can increase the removal rate of MPs and NPs compared to other biological wastewater treatment methods. Studies of MPs removal from wastewater at full industrial or pilot scale in the Netherlands, China, the US, the UK, and Finland have confirmed the effectiveness of MBR, with efficiencies ranging from 64.4 to 99.9% [33,36,70,74,94,156,157,158]. Lares et al. [36] observed that MBR treatment technology reduced the concentration of MPs in raw wastewater from 57,600 MPs/m^3^ to 400 MPs/m^3^ in treated wastewater, achieving higher efficiencies (99.3%) compared to conventional activated sludge. Similar results were obtained by Michielssen et al. [93]. However, it should be emphasized that the removal efficiency of MPs and NPs by the membrane is influenced by many factors, such as their structure and morphological properties, the membrane material and properties, the interaction between the membrane and MPs, the presence of other contaminants in the wastewater, and the susceptibility to membrane fouling [5]. Therefore, different efficiencies of MPs removal by MBR can be found in the literature.

The removal efficiency of MPs in the MBR process is a function of particle size, as the membranes used have pore sizes specific to the MF or UF process and depend furthermore on the type of membrane material [119]. The most common studies are for MPs particles of 100–200 μm in various WWTPs [40,74,159]. Blair et al. [160] showed that WWTPs using advanced purification techniques, including MBR, can effectively remove MPs in the size range of 60–2800 μm. Mintenig et al. [35] reported 95% removal of MPs in the 20–500 μm size range, while Talvitie et al. [89] showed 70% and more than 95% reduction of MPs in the 20–100 μm and 20–300 μm size ranges, respectively. Among all shapes of MPs in WWTPs, fibers dominate, especially in treated effluents, where their content is estimated to be 55–71% [20,36,93,161]. Studies have shown that fibers are more efficiently removed than fragments in pretreatment [89], while the opposite is true in biological treatment [94,162]. Studies have shown that MPs with smaller sizes, especially fibers, are not completely removed by MBR due to the high length-to-width ratio [78,119]. Thus, after filtration, they remain in the sludge, which has to be reprocessed as solid waste, ultimately increasing treatment costs. Li et al. [119] investigated the removal efficiency of a PVC gel with a concentration of 10 MPs/L (particle size < 5 μm) by an MBR with an immersion membrane of 0.1 μm pore size and 0.1 m^2^ surface area. Under conditions of 2.5 h HRT, a temperature of approximately 19.1 °C and pH 7.5, virtually no MPs were detected in the permeate from the MBR system. The results indicated that MPs deposition on the membrane surface could lead to higher fouling, also irreversible. Overall, MBR has a higher capacity to remove all size fractions (especially the smallest sizes in the range 20–100 μm) and all shapes of MPs from wastewater compared to other advanced treatment methods [33]. In addition, compared to other treatment technologies, the shape, size, and composition of MPs appear to have less impact on the removal efficiency of the MBR process.

The paper [84] compared the effectiveness of MBR with other wastewater treatment technologies (disk filter, rapid sand filtration, and dissolved air flotation) in removing MPs (Table 6). An MBR containing 20 submerged UF membranes with flat sheets of 0.4 μm pore size and 8 m^2^ surface area was used. The study showed that most MPs were removed after passing through the MBR system. Compared to other advanced treatment processes, MBR showed a significant improvement in MPs removal (99%), higher final effluent quality, and great potential for reducing the number of process steps, replacing conventional secondary settling tanks in the AS process. A reduction in MPs from 6.9 ± 1.0 MPs/L to 0.005 ± 0.004 MPs/L was achieved [33]. Similarly, Lares et al. [36] obtained 99.4% removal of MPs, indicating that the rate of MPs removal in MBR is consistent and significant. Bayo et al. [156] compared MBR and rapid sand filtration (RSF) technology in the removal of MPs, obtaining average efficiencies of 79 and 75.5%, respectively. Efficiencies for non-fiber forms were higher at 98.83% and 95.53% for MBR and 57.65% and 53.83% for RSF, respectively.

Hybrid systems combining MBR with advanced physical and chemical processes used in water and wastewater technology are more effective in removing MPs. Thus, the MBR-RO hybrid system is an effective and advanced wastewater treatment technology most commonly used to achieve high-quality water [163,164,165]. A five-month pilot study demonstrated the potential of this hybrid process to produce high-quality water directly from municipal wastewater [163]. A comparison was also made between the MBR-RO process and the conventional AS-MF-RO process. The results showed that the MBR-RO process demonstrated the ability to produce similar or better product quality in terms of basic parameters (total organic carbon, NH_4_, and NO_3_) compared to the conventional AS-MF-RO process in municipal wastewater treatment. RO membranes in the MBR-RO process were able to operate at a capacity of 22 L/m^2^h without membrane cleaning for the entire study period, which was 30% higher than in the AS-UF-RO process (17 L/m^2^h) [163]. It was concluded that the MBR-RO process could be a new option in water restoration. Other studies included a comparison of a hybrid MBR system and anaerobic/anoxic/oxygen (A^2^O) treatment in a full-scale wastewater treatment plant in eastern China, with an oxidation ditch (OD) [77]. The influent municipal wastewater contained PET (47%), PS (20%), PE (18%), and PP (15%), with MPs fragments (65%) and fibers (21%) predominating, with a small amount of films (12%) and foams (2%). MPs were removed 99.5% in the MBR- A^2^O system compared to 97% in the OD system on a plastic weight basis, while in terms of the number of MPs the process efficiencies were 82.1% and 53.6%, respectively. The MBR-A^2^O system has a significantly higher MPs removal efficiency than the OD system, probably due to the presence of membrane filtration. MBR-based systems (MBR-A^2^O) effectively remove MPs after pretreatment, retaining virtually all of them in the sludge and blocking their passage to the permeate using a membrane with a pore size <0.1 μm [77]. In addition, MBR in combination with sorption and filtration processes has been shown to be highly effective in removing MPs from WWTPs influent wastewater [166]. Baresel et al. [167] investigated the effectiveness of a hybrid MBR system (with a UF module) and a granular activated carbon biofilter with a total surface area of 0.3 m^2^ for the removal of micropollutants including MPs from real wastewater from the WWTP in Stockholm. It was found that all micropollutants tested, such as pharmaceutical residues, phenolic compounds, bacteria, and MPs particles, could be removed below detection limits or very low concentrations. This demonstrates that the combination of filtration, adsorption, and biodegradation provides extensive and effective removal of micropollutants and the effects of.

Despite the high removal efficiency of MPs and NPs by conventional wastewater treatment processes, advanced treatment, such as MBR, is needed to reduce the amount of these MPs pollutants in the final effluent [35,74,89,156,168]. The MBR process shows the best efficiency of all biological wastewater treatment methods and could become the leading biological method for MPs removal. The MPs removal efficiencies of standard second-stage treatment processes are summarized in Table 7.

Topics for future research should also include the effect of MPs on membrane fouling and the degradation and/or transformation of MPs in MBRs. Other major limitations of MBR technology in wastewater treatment are the control of biofilm thickness, membrane fouling, and liquid distribution, which determine the effectiveness of the method [7,78,169].

#### 3.2.6. Membrane Fouling and Its Impact on the Removal of MPs/NPs

Fouling is one of the main limitations of membrane separation processes, as it reduces the efficiency (permeate flux) in the long and short term and adversely affects the removal efficiency [168,170]. The substances that cause fouling are mainly particulates and colloids, as well as organic and inorganic small- and large-molecule solutes (proteins, carbohydrates, oils, calcium, and magnesium salts (scaling), biological substances (biofouling), and others). Fouling occurs primarily in membrane processes that use porous membranes, i.e., in MF and UF, but to a lesser extent in RO and NF processes, as they generally require intensive pretreatment of wastewater or raw water. The intensity of fouling depends on physical and chemical parameters such as concentration, temperature, pH, ionic strength, and membrane material [126,171,172,173,174,175].

Intermolecular repulsion of MPs and electrostatic interactions between them and the membrane surface are the main mechanisms of MPs removal by membrane filtration [103,175]. In addition, a reduction in filtration efficiency also occurs through the adsorption and deposition of MPs or other substances on the membrane surface [103]. Studies have shown that the permeate flux of a polysulfone UF membrane was reduced by 38% due to the interaction of NPs/MPs with the membrane surface and pores [112]. The mechanism of membrane fouling with MPs particles is initially indirect and may eventually lead to complete blocking of the membrane pores. Filter cake formation and blocking of the internal pores of the membrane then occur, which are considered to be the end result of membrane fouling with MPs particles [176,177,178]. It is also important to understand the mechanism of fouling of membrane systems by NPs in order to determine their effect on filtration performance. Previous studies focusing on particulate filtration have shown flux reductions ranging from 47% to 79% after filtration with SiO_2_ (25 nm), TiO_2_ (21 nm), ZnO (35 nm) nanoparticles [179], and bentonite clay (4.6 μm) [180], with low permeate flux recovery rates after membrane cleaning [181]. In addition, NPs have irregular shapes after fragmentation [178,182], which can lead to membrane damage by the edges of NPs [178]. This phenomenon is particularly evident in RO membranes where high pressures are applied [103].

Due to their ability to completely or partially block the pores, MPs of small sizes induce a greater fouling effect than large ones [97,113]. In studies of UF (60,000 kDa MWCO membrane) latex with PS (0.2–200 mm), membrane pore blocking was the main fouling mechanism at particle sizes smaller than 10 mm [182]. This is likely to be due to the attractive forces between the particles and the membrane surface, leading to 40% irreversible fouling. On the other hand, particles larger than 10 mm became fouling agents by depositing a porous cake layer (70%) instead of blocking the pores of the (30%).

Although NPs/MPs present in water are expected to contribute to membrane fouling during water treatment, the actual impact of NPs/MPs on membrane filtration performance remains unknown [22]. It is therefore crucial to clarify the mechanisms of membrane fouling by NPs/MPs in order to develop appropriate filtration and cleaning procedures that maintain membrane systems at high performance levels. In addition, biofilms forming on the membrane, but also on MPs or other adsorbates, can cause internal, irreversible fouling that shortens membrane life and increases operating costs [112,183].

The following processes are mentioned as standard methods to prevent and reduce fouling by NPs and MPs particles [184]: pretreatment of the feed prior to entering the membrane modules, modification of membrane properties, membrane cleaning (physical and chemical), and optimization of membrane operation conditions. Chemical methods of membrane cleaning usually include acidic, alkaline, or enzymatic solutions, while physical methods include backwashing or passing a mixture of water and air over the membrane surface in the opposite direction to the flow during filtration [176]. Other ways of reducing membrane fouling caused by MPs and NPs are currently being investigated. Enfrin et al. [185] investigated the effect of gas scrubbing as a physical cleaning to mitigate MPs/NPs fouling. Their results showed that gas scrubbing can mitigate MPs/NPs fouling of hydrophobic membranes, while it does not affect fouling of hydrophilic membranes. Another study by the same group [186] investigated the evaluation of MPs/NPs fouling mitigation by plasma treatment, which resulted in less fouling of modified hydrophilic plasma-coated membranes. Li et al. [118] used the addition of aluminum-based flocs (Al-flocs) in the presence of MPs, leading to mitigation of fouling caused by the formation of loose layers of the filter cake of UF membranes. Wang et al. [187] studied the effect of pre-chlorination on the removal of MPs by the membrane. The results showed that the addition of an oxidant (chlorine) reduces the deposition of MPs from the PS on the membrane surface, decreases the permeate flux decline rate by 15.1%, and decreases the MPs removal rate from 36.6% to 22.6%.

A study of the effect of MPs on membrane biofouling in the UF process of lake water was also carried out [188], which showed an increase in microbial activity in the presence of MPs. MPs stimulated the production of EPS (polysaccharides and proteins), resulting in an increase in the abundance of *Alphaproteobacteria*, *Flavobacterium*, and *Pseudomonas*, which accumulated on the membrane surface, causing biofouling. Other studies of the effect of MPs on the UF process confirmed not only increased microbial growth but also influenced the type of microorganisms (e.g., *Xanthobacteraceae*, *Sphingomonadaceae*, *Leptolyngbyaceae*) that can promote EPS production and nitrogen fixation, causing rapid membrane biofouling [189]. The presence of MPs may therefore promote the microbial activity of some microorganisms that are capable of biodegrading plastics and chemicals added to plastics. The above studies have shown a close relationship between MPs, membrane biofouling, and the potential adverse effects of MPs on wastewater treatment and drinking water treatment.

In general, the presence of MPs increases the intensity of membrane fouling; therefore, more systematic studies on the effect of MPs on fouling and possible methods to mitigate it are warranted.

## 4. Recycling and Reuse of Polymeric Membranes

Currently, polymeric membranes are most commonly used in wastewater treatment plants and, as a result, are more susceptible to damage from NPs in the water [190]. According to the latest data from Global Info Research, the global membrane filtration market size was valued at 6765.3 million USD in 2022 and is projected to reach an adjusted size of 10,000 million USD by 2029. The largest number of the industry’s products worldwide come from the US and Western Europe, with around 25% of the market share held by two companies, namely SUEZ (GE Water) and DowDuPont [191].

It is estimated that more than 840,000 end-of-life RO membrane modules (>14,000 tons of plastic waste) are sent to landfill each year worldwide [192]. For these reasons, it is suggested that the management of end-of-life membranes should be transferred to a closed-loop economy. The closed-loop economy is the most promising way to use plastics sustainably [193]. It aims to reduce their consumption by keeping materials in the value chain for longer periods of time compared to the traditional linear flow of materials. In addition to reducing plastic consumption, reducing or eliminating plastic pollution (including MPs and NPs pollution) is the second main objective of the circular economy. However, studies on the transition to a closed-loop economy rarely consider this second aspect [193]. Achieving these objectives will require a number of actions, which include, but are not limited to, the following [192,193,194]:

(1) Innovation, development, and use of new polymers with improved durability compared to existing single-use products and with reusability and recyclability.
(2) The elimination or significant reduction in the passage of MPs and NPs into the environment throughout the life cycle of a given product, and new-generation polymers should have a significantly shorter time to return to the environment compared to existing polymers.
(3) Efforts to recycle plastic products must not result in an increase in the release of MPs and NPs into the environment, and recycling activities should be the responsibility of the producer.

The widespread use of membrane processes has spawned the need to develop methods to reuse and recycle used membrane modules [195]. RO membranes are the largest type of practical application, accounting for 42% of the total membrane filtration market. Studies have shown that almost 70% of used membranes are recyclable and that reuse saves between 85% and 95% of energy compared to purchasing new commercial membranes. These studies concern the manufacture of membranes for UF and NF from recycled used RO membranes using various chemical treatments, such as oxidation with sodium hypochlorite (NaClO) [192,196]. The effectiveness of such membranes has been confirmed at laboratory and pilot scale in desalination and wastewater reclamation applications [192]. There is also a company based in Germany that recycles and reuses membranes at the end of their life [197]. In cases where used membranes are excessively damaged and exhibit too low a retention rate for UF and NF purposes, a better solution is the so-called intermediate recycling, which involves separating plastic components from the RO module for individualized use [195]. An interesting intermediate recycling solution is the fabrication of ion exchange membranes (IEMs) for electrodialysis (ED) using spent RO membranes as a support layer [195]. Studies included the effect of preparation conditions on membrane properties. In addition, the desalination potential of brackish water in a laboratory ED system was evaluated, comparing the results obtained with commercial ED membranes. Furthermore, polypropylene (PP) RO module spacers can be reused as turbulence promoters (spacers) between membranes or compartments in the ED module [196].

Furthermore, it should be noted that membrane production is increasingly oriented towards the use of new bio-based (recyclable and biodegradable) polymers as an alternative to petrochemical polymers [198].

## 5. Concluding Remarks

MPs and NPs represent a serious global pollution problem for our planet, comparable to global warming, as millions of tons of these pollutants enter the aquatic environment every year through raw and treated municipal and industrial wastewater. An analysis of the literature shows that conventional wastewater and natural water treatment methods are not able to completely remove MPs and NPs from drinking water and wastewater. Efficient processes must therefore be developed to eliminate them from natural waters and both municipal and industrial wastewater. Filtration is considered the most effective physical method for the removal of MPs, although further work is still needed on its implementation in large-scale wastewater treatment. Hydrophytic and MBR technologies are effective among biological treatment methods. In chemical treatment, coagulation, and electrocoagulation, Fenton methods show promising results in removing MPs.

One of the advanced technologies for tertiary wastewater treatment and drinking water treatment appears to be membrane processes, which are characterized by high efficiency in the removal of MPs and NPs. The main mechanism of membrane separation is based on exclusion by size (sieve mechanism), which offers great potential for adjusting the membrane pore size and thus selecting the type of process to suit the particle size of MPs/NPs. In addition, other mechanisms, including hydrophobic and electrostatic interactions, affect their removal efficiency by modifying the membrane surface properties and MPs/NPs, changes in pH, and other properties. Therefore, the use of pressure-driven membrane processes, i.e., MF, UF, NF, and RO, in DWTP and WWTP reduces the concentration of MPs/NPs in wastewater or drinking water. In this way, membrane technologies allow fewer MPs/NPs to enter tap water and wastewater from treatment plants, and thus the environment, than with other technologies.

Hybrid technologies for the removal of MPs or NPs, such as MBR or combined coagulation and membrane filtration, appear to be the most effective means of removing these pollutants. In field tests at wastewater treatment plants, MBR showed the highest removal rate, at 99.9%, of the other treatment methods tested (e.g., RO, sedimentation, flotation, and RSF). Advanced hybrid systems such as the MBR-UF/RO system, coagulation followed by ozonation, granular activated carbon, dissolved air flotation, filtration, and hybrid technologies based on hydrophytic treatment also show very promising results in the effective removal of MPs.

UF combined with a coagulation step is one of the main water treatment technologies in current water utilities, showing significant removal of organic matter, including MPs. Systematic studies have shown that the efficiency of MPs removal in coagulation and UF processes has great potential for full application in drinking water treatment.

Prospects for future research:

(1) Efforts to develop and refine hybrid methods for MPs/NPs removal should be increased, especially the degradation and/or transformation of MPs in MBR should be investigated.
(2) The number of studies on the removal of MPs and NPs in DWTP and WWTP under full industrial scale conditions should be increased, as most studies are carried out under controlled conditions in the laboratory or on a pilot scale, whereas under real conditions there is a high probability of reduced efficiency.
(3) Alternative methods to prevent membrane fouling due to MPs/NPs should be developed, and the focus should be on producing membranes with MPs/NPs anti-fouling and self-cleaning properties.
(4) There is a need for more research in the future on the use of inorganic material membranes in the removal of MPs/NPs. Currently, polymeric membranes are more widely used in DWTP and WWTP than inorganic membranes due to their low cost and ease of manufacture.
(5) During polymeric membrane processes, there is a possibility of MPs/NPs release (attrition) into water/wastewater not only through porous membranes but even through dense osmotic membranes such as for RO. Further research is needed on the conditions for the release and permeation of MPs from polymeric membranes into water/wastewater and how to minimize this phenomenon.
(6) There is also a paucity of research on MPs passing into retentate (concentrate), even though membrane filtration of MPs is highly effective.
(7) Actions concerning the elimination or reduction in MPs/NPs pollution, which can act simultaneously, include mainly raising public awareness of pollution policies, limiting the use of single-use plastics and banning plastics in personal care products, and implementing processes based on the use of biodegradable materials.

## Figures and Tables

**Table 1:** Presence of MPs in surface water from different countries.

Source	Country	Surface water (m^−3^)	Sediment (kg^−1^)	Dominant MPs
Jangcy River, Shores, and Island Chongming	China	0–259	10–60	Polyethylene, polypropylene, α-cellulose
Tampa Bay	USA	940	30–790	No data available
The Baltic coast	Germany	0–5000	No data available	No data available
Drinking water treatment plants	Germany	0–7	No data available	Polyethylene, polyamide, polyester, polyvinyl chloride
Stream, river, and lake	USA	0.06–19.10	32.9–6229	Polyethylene, polypropylene, polyethylene terephthalate
Seine	Francee	3–108	No data available	Fibers
Rivers	USA	1.94–17.93	No data available	Fibers
Great Lakes	USA	0.05–32	No data available	Fibers
Surface water and sediments	Hong Kong	51–27,909	49–279	Polypropylene, polyethylene, ethylene, propylene, styrene, acrylonitrile
Venetian lagoon	Italy	No data available	672–2175	Polypropylene, polyethylene

Source: own elaboration based on references [20,21,27,28,29,30,31,32].

**Table 2:** MPs removal by different stages of wastewater treatment plants.

Purification Process	Removal of MPs (%)	Location of WWTP
I treatment stage/AS	99.9	Sweden
I treatment stage/AS	88.1	France
I treatment stage/AS	99.9	USA
I treatment stage/AS	98.4	Scotland
I treatment stage/AS	11–94	Netherlands
I treatment stage/AS	95.6	USA
I treatment stage/AnMBR	98.3	Finland
I treatment stage/MBR	99.4	Finland
I, II, and III treatment stage (GF)	99.3	USA
I, II, and III treatment stage (BAF)	97.8	Finland

Source: own elaboration based on references [33,94].

**Table 3:** Comparison of pressure-driven properties of membrane processes.

Microfiltration	Ultrafiltration	Nanofiltration	Reverse osmosis
Particle separation (e.g., bacteria and viruses)	Separation of high-MW and colloidal substances (e.g., proteins)	Separation of multi-valent ions and organic compounds with MW > 300	Separation of low-MW substances (e.g., salts)
Osmotic pressure—may be omitted	Osmotic pressure—may be omitted	Osmotic pressure—plays a role	High osmotic pressure: 0.5–2.5 MPa
Low TMP (<0.2 MPa)	Low TMP (0.1–1.0 MPa)	The TMP is 0.5–2.0 MPa	High TMP (1.0–6.0 MPa)
Symmetric membrane structure	Asymmetric membrane structure	Asymmetric membrane structure	Asymmetric membrane structure
Thickness of the separation layer (epidermal): 10–150 μm.	Thickness of the separation layer: 0.1–1.0 μm	Thickness of the separation layer: 0.1–1.0 μm	Thickness of the separation layer: 0.1–1.0 μm
Separation mechanism—sieve	Separation mechanism—sieve	Dissolution and diffusion separation	Dissolution and diffusion separation

Source: own elaboration based on references [101,102,103,104,105].

**Table 4:** Comparison of MPs removal efficiencies from PA and PS by polycarbonate, cellulose acetate, and polytetrafluoroethylene membranes.

Membrane	MPs	Concentration MPs [1/L]	Medium Size of MPs [μm]	Removal Efficiency [%]
PC	PA	127,000	15.66	99.6
	PS	33,000	37.40	96.8
CA	PA	27,000	20.58	99.8
	PS	8000	75.51	94.3
PTFE	PA	46,000	21.72	99.6
	PS	47,000	29.49	96.0

Source: own elaboration based on reference [113].

**Table 5:** Retention rates of NPs with PS.

Membrane Material	Characteristics of PS		PS Retention (%)
	Type	Size	
UF—regenerated cellulose: 30 kDa	PS 120	120 nm	100
	PS 500	500 nm	100
	BSA	66 kDa	-
	PS 120 + BSA	Mixture	100
	PS 500 + BSA	Mixture	100
UF—polyethersulfone: 30 kDa	PS 120	120 nm	100
	PS 500	500 nm	100
	BSA	66 kDa	-
	PS 120 + BSA	Mixture	100
	PS 500 + BSA	Mixture	100
MF—chlorinated polyethylene: 0.4 μm	PS 120	120 nm	26.72
	PS 500	500 nm	100
	BSA	66 kDa	-
	PS 120 + BSA	Mixture	0
	PS 500 + BSA	Mixture	100

Source: own elaboration based on reference [117].

**Table 6:** Average MPs concentrations before and after treatment with different technologies.

Treatment Technology	Raw Wastewater (MPs/L)	Treated Wastewater (MPs/L)	Removal (%)
Disk filter: 10 µm as a third stage of treatment	0.5	0.3	40.0
Disk filter: 20 µm as at third stage of treatment	2.0	0.03	98.5
Rapid sand filter as a third stage of treatment	0.7	0.02	97.1
Flotation (airborne) as a second stage of treatment	2.0	0.1	95.0
MBR	6.9	0.005	99.9

Source: own elaboration based on reference [84].

**Table 7:** Efficiency of MPs removal with different biological treatment methods.

Treatment Type	Effectiveness (%)	Type of MPs in Wastewater
MBR, AS, and settling tank	83.1–91.9	Fragments
AS and clarification	92	Fragments, fibers
AS	93.8	Microgranules
AS	89.8	Microgranules
MBR	79.01	Fibers, PP, PS
A^2^O	71.67 ± 11.58	No data available
AS, sedimentation	64	Fibers
MBR	99	Fragments, fibers from PVC
Hydrophytic treatment plant	97	Fragments, fibers
AS	52	PE < 100 µm
Aerated biological filter	99	PE100–300 µm
A^2^O	54.4	-
A^2^O	28.1	PET, PE, PES, PAN, PAA
AS	66.7	PS
MBR	99.9	20–100 μm MPs
MBR	97.6	PES fibers and PE fragments
A^2^O	93.7	PE, PP, PE
MBR	99.4	PES, PE, PA, and PP
AS	98.3	Different types of MPs
AS	75–91.9	Different types of MPs
Submerged MBR	100.0	
Submerged anaerobic MBR	99.4	
Submerged MBR (KUBOTA)	100	

Source: own elaboration based on references [33,77,89,93,136,159,160,168,169,170,171,172,173,174].

## Data Availability

No new data were created or analyzed in this study.

## Abbreviations

A^2^O	anaerobic-anoxic-oxygen
AnMBR	anaerobic membrane bioreactor
AS	activated sludge
BAF	biologically active filter
BOD	biochemical oxygen demand
BSA	bovine serum albumin
CA	cellulose acetate
DS	draw solution
DWTPs	drinking water treatment plants
ED	electrodialysis
FDFO	fertilizer-driven forward osmosis
FO	forward osmosis
FS	feed solution
GF	granular filter
HRT	hydraulic retention time
IEMs	ion exchange membranes
IMS	integrated membrane system
MBR	membrane bioreactor
MF	microfiltration
MPs	microplastics
MW	molecular-weight
NF	nanofiltration
NPs	nanoplastics
O_3_	ozone
OD	oxidation ditch
P	pressure
PA	polyamides
PAHs	polycyclic aromatic hydrocarbons
PAM	polyacrylamide
PAN	polyacrylonitrile
PC	polycarbonate
PCBs	polychlorinated biphenyls
PE	polyethylene
PES	polyethersulfone
PEs	polyester
PET	polyethylene terephthalate
PFASs	per-/polyfluoroalkyl substances
PP	polypropylene
PS	polystyrene
PTFE	politetrafluoroetylen
PVC	polyvinyl chloride
RC	regenerated cellulose
rGO	reduced graphene oxide
RO	reverse osmosis
RSF	rapid sand filter
SR	synthetic rubber
SRT	sludge retention time
TMP	transmembrane pressure
UF	ultrafiltration
USA	the United States of America
UV	ultraviolet
WWTPs	wastewater treatment plants
